# Bayesian Activity Estimation and Uncertainty Quantification of Spent Nuclear Fuel Using Passive Gamma Emission Tomography

**DOI:** 10.3390/jimaging7100212

**Published:** 2021-10-14

**Authors:** Ahmed Karam Eldaly, Ming Fang, Angela Di Fulvio, Stephen McLaughlin, Mike E. Davies, Yoann Altmann, Yves Wiaux

**Affiliations:** 1 Institute of Sensors, Signals and Systems, School of Engineering and Physical Sciences, Heriot-Watt University, Edinburgh EH14 4AS, UK; s.mclaughlin@hw.ac.uk (S.M.); y.wiaux@hw.ac.uk (Y.W.); 2 Department of Nuclear, Plasma, and Radiological Engineering, University of Illinois at Urbana-Champaign, Urbana, IL 61801, USA; Mingf2@illinois.edu (M.F.); difulvio@illinois.edu (A.D.F.); 3 Institute for Digital Communications & The Joint Research Institute for Signal and Image Processing, The University of Edinburgh, Edinburgh, EH9 3JL, UK; Mike.Davies@ed.ac.uk

**Keywords:** inverse problems, imaging, Bayesian inference, uncertainty quantification, tomography, Markov chain Monte Carlo

## Abstract

In this paper, we address the problem of activity estimation in passive gamma emission tomography (PGET) of spent nuclear fuel. Two different noise models are considered and compared, namely, the isotropic Gaussian and the Poisson noise models. The problem is formulated within a Bayesian framework as a linear inverse problem and prior distributions are assigned to the unknown model parameters. In particular, a Bernoulli-truncated Gaussian prior model is considered to promote sparse pin configurations. A Markov chain Monte Carlo (MCMC) method, based on a split and augmented Gibbs sampler, is then used to sample the posterior distribution of the unknown parameters. The proposed algorithm is first validated by simulations conducted using synthetic data, generated using the nominal models. We then consider more realistic data simulated using a bespoke simulator, whose forward model is non-linear and not available analytically. In that case, the linear models used are mis-specified and we analyse their robustness for activity estimation. The results demonstrate superior performance of the proposed approach in estimating the pin activities in different assembly patterns, in addition to being able to quantify their uncertainty measures, in comparison with existing methods.

## 1. Introduction

In order to deter the proliferation of nuclear weapons, safeguards provide various technical measures that are used for the verification and the declarations made by the signatories to the Treaty on the Non-Proliferation of Nuclear Weapons, regarding their nuclear material and activities [1]. An important task within these safeguards is monitoring of spent fuel assemblies (SFAs) from nuclear power plants (NPPs), for detecting any eventual diversion of spent nuclear fuel for non-declared purposes. The detection of a single fuel pin missing from SFA should be reported. For any safeguards investigation of SFAs, it is important to use a minimum amount of a priori information on the SFA under study, in order to avoid biasing and potentially misleading the investigation. Eventually, IAEA approved the use of the passive gamma emission tomography (PGET) instrument [2,3,4,5,6,7] in inspections.

Passive gamma emission tomography is an imaging modality that can be used for the verification of spent nuclear fuel stored in water pools [4]. Spent nuclear fuel is highly radioactive; hence, the tomography can be conducted in passive mode, without the need of an external X-ray source, as in traditional tomography. The detection unit considered in this work consists of two collimated CdTe detector arrays, on opposite sides of the fuel assembly, each encompassing 91 detectors. The detector arrays rotate and scan the fuel bundle in steps of one degree, which generates a sinogram by detecting gamma rays emitted by the fuel pins. A cross-sectional image of the spent fuel bundle, using image reconstruction algorithms, can then be reconstructed. Based on these images, the fuel pins can be identified and their activity estimated as the sum of pixel values inside each pin region. Image reconstruction techniques with the capability of estimating the fuel pins’ activity accurately are crucial in nuclear safeguards, as they allow inspectors to monitor nuclear material and promptly identify its diversion.

Due to the nature of acquisition process (e.g., the discrete nature of the measured photon counts), the observation noise of PGET sinograms is expected to be well-approximated by Poisson noise. However, if the photon counts in a sinogram pixel is high, the Poisson distribution becomes more symmetric, and it can be well-approximated using a Gaussian distribution, whose mean and variance change across pixels. While the sinogram formation process can be approximated by a linear forward model (which simplifies the inversion), this first-order approximation does not take into account the attenuation of the measured radiation of a pin, due to the presence of other pins (or shielding materials) within the assembly. Consequently, the activity levels, estimated by inversion of a linear problem, can present biases and artefacts, depending on the noise model considered. Hence, in this work, we provide a comparison of both the Poisson and isotropic Gaussian noise models and investigate their robustness. Moreover, we also investigate different linear operators corresponding to different levels of prior knowledge about the assembly configuration.

Most of state-of-the-art algorithms considered to solve linear inverse image restoration problems (irrespective of the noise model) are either optimization or simulation-based methods. Optimization-based approaches primarily rely on log-concave Bayesian models, such as [8,9,10,11,12,13,14,15], and have been proposed to perform maximum-a-posteriori (MAP) estimation. For example, PIDAL, which stands for *Poisson image deblurring using augmented Lagrangian* [8], and SALSA, which stands for *split augmented Lagrangian shrinkage algorithm* [10], are Poisson and Gaussian image restoration algorithms based on a total-variation loss ot sparsity-promoting prior, which solves the restoration problem using an alternating direction method of multipliers (ADMM). Moreover SARA, which stands for *sparsity averaging reweighted anlysis* [11,12], assumes that the ill-posed problem is regularized by the assumption of average signal sparsity over representations in multiple wavelet bases. Although such algorithms are efficient in computing maximum *a posteriori* (MAP) estimates relatively quickly, they cannot provide uncertainty maps for the estimates, which can be very valuable in applications involving subsequent decision making, such as pin identification and activity estimation in the PGET context. Alternatively, many studies have considered hierarchical Bayesian models to solve the deconvolution and restoration problems akin to those in [16,17,18]. This class of methods assumes that the unknown model parameters are unknown stochastic quantities by assigning them suitable prior distributions, based on prior beliefs. The joint posterior distribution can then be computed using Bayes theorem and exploited using Markov chain Monte Carlo methods. These models offer a flexible and consistent methodology to deal with uncertainty in ill-posed inverse problems. Moreover, additional unknown parameters can be jointly estimated within the algorithm, such as regularization parameters. As such, they represent an attractive way to tackle ill-posed problems, such as the one considered in this work, where an automated hyperparameter setting is challenging. As an example, the authors in [17] proposed a Bayesian approach that samples from a posterior distribution built on a Poissonian likelihood and standard convex and possibly non-smooth regularizers. That study proposed an approach that relied on the split-and-augmented Gibbs sampler (SPA) [18] to tackle a Poisson/Gaussian reconstruction problem by sampling from an approximate joint posterior distribution. In particular, the log-concave property of the prior distributions allows the derivation of log-concave conditional distributions that are decoupled from the likelihood and can be sampled by proximal MCMC methods [19,20]. In this context, the SPA method provides both image estimates, such as MAP and MMSE, and their corresponding posterior uncertainty measures.

The PGET instrument is essentially a single photon emission computed tomography (SPECT) system that allows the reconstruction of axial cross-sections of the emission map of the SFA. Industrial or medical tomographic imaging systems work in a similar manner as PGET systems, in terms of data acquisition. Furthermore, the PGET concept can be applied to perform fast neutron imaging by replacing gamma ray detectors with neutron detectors [21]. However, the PGET of spent fuel poses some unique challenges, due to the high activity of the sources (a single-pin activity is of the order of $10^{13}$ Bq) and the high self-attenuation of the fuel pins. Hence, robust image reconstruction algorithms are crucially required. For instance, in [7], the authors proposed a method to simultaneously reconstruct the activity and attenuation maps by formulating the reconstruction as a constrained minimization problem with a least squares data fidelity term and quadratic regularization terms with different smoothness matrices. However, the forward model proposed in [7] considered only monoenergetic gamma rays and did not account for the scattering effects of gamma rays in the PGET system that make non-neglible contributions to the total counts, which added additional uncertainty to the activity estimation results. Similarly, in [22], the authors formulated the problem of pin activity estimation as an optimization problem where a sparse regularization function is used. Although the advantages of these methods in providing fast estimates, they can provide only point estimates; hence, they can neither quantify the uncertainty of the estimates nor provide probability of presence of the pins. Moreover, associated regularisation parameters need to be manually tuned by the user, which can affect the estimated activity levels. In this work, to improve the quality of the estimated activity profiles and reduce the computational cost of the inversion procedure, we propose to only estimate the activity in a reduced number of pixels, leveraging the prior knowledge that the activity profile is expected to be null outside pin locations. For instance, for a cross-sectional image of the field of view discretized into 182×182 pixels, the pins, whose maximum number is known, form groups of 10 to 16 connected pixels, which form a small subset (≈15%) of the $182^{2}$ pixels. The method proposed in this paper assumes that a mask, identifying where pins are expected to be present, is available. This mask is used (i) to account for pin self-attenuation when building the linear approximate forward operator and (ii) as an upper bound for the support of the pins present in the assembly. While the geometry of the assembly is generally known, the exact presence map is often unknown (this is one feature we want to estimate), but several initial guess can be considered, as will be discussed in Section 6. The main contributions of this work can be summarized as follows.

- We formulate the pin activity estimation problem within a Bayesian framework and assign a Bernoulli truncated-Gaussian (BtG) prior model to the intensity field to be estimated. To the best of our knowledge, no work attempted to sample from such a highly-multimodel joint posterior distribution using an SPA sampler. This allows for estimating the activity of spent-fuel, including the assessment of fuel rod presence/absence.
- We compare the performance of two different noise models for pin activity estimation from PGET sinograms simulated while accounting for pin self-attenuation (i.e., not simulated using a linear model).
- In addition to estimating the activity profile, the proposed algorithms allow the automated estimation of the crucial model hyperparameters, like regularization parameters, which might affect the resulting estimated activity.

The remaining sections of the paper are organized as follows. Section 2 formulates the problem of PGET image restoration, followed by Section 3, which summarizes the likelihoods and the prior distributions assigned to the unknown parameters of the models. The resulting joint posterior distribution and the partially collapsed Gibbs sampler, used to sample that distribution, are discussed in Section 4. Simulations conducted using synthetic data (following a linear models used for inversion) are presented in Section 5 and the analysis of more realistic data is discussed in Section 6. Conclusions and future work are, finally, reported in Section 7.

## 2. Problem Formulation

**Figure 1:**
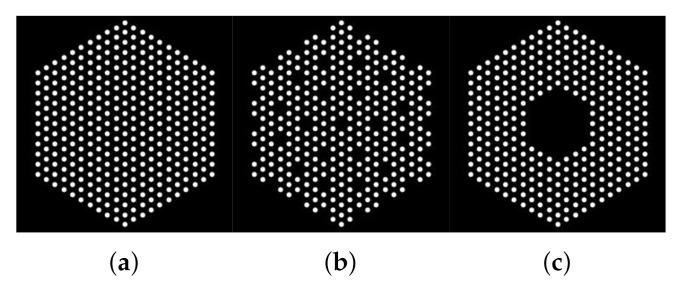
Examples of binary masks of the simulated fuel assemblies. (**a**) Case “1”, (**b**) Case “2”, and (**c**) Case “3”. In Case “1”, the mock-up fuel assembly hosted 331 ${}^{60}$Co pins of 8.879 g/cm${}^{3}$ density, which emitted 1.17 and 1.33 MeV gamma rays. In Cases “2” and “3”, 10% ${}^{60}$Co pins pins were removed at the center or uniformly.

Figure 1 shows examples of different pin configurations (i.e., presence maps) akin to those expected to be studied using a PGET system. We can observe that each cross-sectional image consists of pins formed by groups of connected pixels (white), which will emit radiations. Black pixels do not emit measurable radiations. In this work, we assume that we know, *a priori*, a mask such as those in Figure 1 such that the activity of the black pixels is assumed to be null and thus not estimated. However, the mask can be designed conservatively and select pixels where no pins are present (e.g., the mask in Figure 1a can be selected although the actual assembly configuration is that depicted in Figure 1b). Using this used-defined mask, we defined as $x={\left(x_{1},\cdots ,x_{N}\right)}^{T}\in R^{N}$, the concatenation of the pixel values to be estimated.

The pin activity estimation problem is formulated as follows. Given a set of measurements (sinogram) $y={\left(y_{1},\cdots ,y_{M}\right)}^{T}\in R^{M}$, we aim at recovering the underlying pin intensities $x={\left(x_{1},\cdots ,x_{N}\right)}^{T}\in R^{N}$, which are related to the observation y by y=G(x)+w, where G is a function representing the system’s response, and w represents random observation noise. Due to photon scattering and attenuation effects between pins during the data formation process, the function G is non-linear and difficult to model analytically. However, it can be approximated by a linear model based on the user-defined mask discussed above. Indeed, the presence map can be used to build a linear mapping from x and *y* whereby attenuation involving neighbouring pins is accounted for (the attenuation is mostly due to the presence of pins, not their activity). Hence, in this work, the function G is approximated by a linear operator $A\in R^{M\times N}$, which is built using the simulator recently considered in [22]. Consequently, the forward model considered in this work can be expressed as:

(1) $$\begin{array}{c} y|x∼F(Ax), \end{array}$$

where F(·) denotes a probability distribution and ∼ reads “is distributed according to”. While the observation noise corrupting PGET sinograms is expected to be Poisson distributed, we will consider two different noise models as the linear approximation can introduce mis-modeling errors. The problem investigated in this paper is the estimation the pin intensities x from the observation vector y. This inverse problem is severely ill-conditioned because of A and prior regularization is necessary to promote the solution to be in a set of feasible intensities x. To solve this problem, we propose a hierarchical Bayesian model and a sampling method to estimate the unknown model parameter.

## 3. Hierarchical Bayesian Model

This section introduces the hierarchical Bayesian model proposed to estimate the unknown parameter x. This model is based on the likelihood function of the observations and on prior distributions assigned to the unknown parameters, i.e., x and potential hyperparameters.

### 3.1. Likelihood

Under the Poisson noise model assumption, each individual observation $y_{m},\;m\in \left[1,M\right]$ corresponds to an independent realization of a Poisson random variable, that is, for all m∈[1,M]:

(2) $$\begin{array}{c} f\left(y_{m}|x\right)∼P\left({\left\{Ax\right\}}_{m}\right),\;m=1,\ldots ,M, \end{array}$$

where P(·) denotes the Poisson distribution and ${\left\{Ax\right\}}_{m}$ denotes the *m*th element of Ax. The full likelihood reduces to $f\left(y|x\right)=\prod_{m=1}^{M}f\left(y_{m}|x\right)$.

The second model considered assumes independent and identically distributed (idd) additive Gaussian noise. In that case, the full likelihood reduces to:

(3) $$\begin{array}{c} f\left(y|x\right)={\left(\frac{1}{2\pi \sigma^{2}}\right)}^{N/2}\exp\left(-\frac{{\left\|y-Ax\right\|}_{2}^{2}}{2\sigma^{2}}\right), \end{array}$$

where $\sigma^{2}$ is the (unknown) noise variance.

### 3.2. Prior Distributions

As mentioned earlier, $x\in R^{N}$ is a concatenation of pixel intensities. If the user-defined mask is perfect, then the entries of x are expected to be positive, as long as a pin present has a measurable activity level. However, if the mask is designed conservatively, a pin which is assumed to be present can have a null activity level in practice, i.e., a fraction of the elements of x are equal to 0. In order to model such belief while ensuring the positivity of the activity levels, a classical choice is the following exponential prior distribution:

(4) $$\begin{array}{c} f\left(x|\beta \right)=\frac{\beta }{2}\exp\left(-\beta \|x_{1}\|\right)\times 1_{R^{+}}\left(x\right), \end{array}$$

where β is a regularization parameter controlling the mean value of x and $1_{R^{+}}\left(x\right)$ is the indicator function defined on the positive orthant of $R^{N}$. The resulting joint posterior distribution f(x|y,β) (for a fixed β) is relatively easy to sample from using an SPA sampling strategy, as in [17,18], resulting in an algorithm that is referred to as SPA-P-ℓ1 (Split and Augmented Gibbs sampler for Poisson noise) and SPA-G-$\ell_{1}$ (Split and Augmented Gibbs sampler for Gaussian noise), assuming $\sigma^{2}$ is known. Moreover, one can maximize the logarithm of the resulting joint posterior distribution and compute the maximum *a posteriori* (MAP) estimates as in [8], resulting in the PIDAL-ℓ1 algorithm using a Poisson image model and FISTA-$\ell_{1}$ for Gaussian image model.

However, in this work, to model the intensity field vector sparsity, we introduce:

(5) $$\begin{array}{c} x_{g}=z_{g}t_{g}, \end{array}$$

where $z_{g}\in \left\{0,1\right\}$ is a binary label for pin *g* and $t_{g}=\left[t_{1}^{\left(g\right)},\ldots ,t_{K_{g}}^{\left(g\right)}\right]\in R^{K_{g}}$ is the corresponding amplitude vector for the $K_{g}$ pixels forming pin *g*, $x_{g}\in R^{K_{g}}$ and $x=[x_{1},\ldots ,x_{G}]$, where *G* is the maximum number of pins in the assembly, as defined by the user-defined mask. If $z_{g}=0$, the activity of any pixel of the pin *g* is 0. If $z_{g}=1$, then $x_{g}=t_{g}$. Note that $K_{g}$ and *G* are known a priori, however, the number of active pins forming a specific assembly (i.e., the value of ${\left\{z_{g}\right\}}_{g}\in {\left\{0,1\right\}}^{G}$) is not known and must be estimated. The decomposition described above allows one to decouple the location of the sparse components from their values. In a similar fashion to [23], to model the sparsity of x, we utilize a structured spike-and-slab prior, which stems from classical spike-and-slab priors that have been used in Bayesian regression and factor models [24,25,26,27]. Following this, we assume that the intensities in ${\left\{t_{g}\right\}}_{g}$ are drawn independently from the same truncated zero-mean Gaussian distribution, i.e.,

(6) $$\begin{array}{c} f\left(\left[t_{1},\cdots t_{N}\right]|s^{2}\right)=\prod_{n=1}^{N}N_{R^{+}}\left(t_{n};0,s^{2}\right), \end{array}$$

where $s^{2}$ controls the prior variance of the intensity vector. The pin presence labels are assumed *a priori* mutually independent and assigned a shared Bernoulli prior distribution, leading to:

(7) $$\begin{array}{c} f\left(z_{g}|\omega \right)=\omega^{z_{g}}{\left(1-\omega \right)}^{\left(1-z_{g}\right)},\;z_{g}\in \left\{0,1\right\}, \end{array}$$

and

(8) $$\begin{array}{c} f\left(z|\omega \right)=\prod_{g=1}^{G}f\left(z_{g}|\omega \right). \end{array}$$

The prior models of t and z depend on the hyperparameters $s^{2}$ and ω which can be difficult to fix automatically. Thus, we include them within the inference process using hyperpriors. To reflect the lack of prior knowledge about the variance $s^{2}$ in Equation (6), a weakly informative inverse-gamma prior is assigned to $s^{2}$:

(9) $$\begin{array}{c} s^{2}∼IG\left(s^{2};\eta ,\nu \right), \end{array}$$

where (η,ν) are fixed to $\left(\eta ,\nu \right)=\left(10^{-3},10^{-3}\right)$. Similarly, we assign ω a conjugate beta prior distribution:

(10) $$\begin{array}{cc} \; & \omega ∼Be(\omega ;\alpha ,\beta ), \end{array}$$

where (α,β) is fixed to (α,β)=(1,1).

Note that we did not observe significant changes in the results when changing the hyperparameters (η,ν) to $\left(10^{-3},10^{-3}\right)$, $\left(10^{-2},10^{-2}\right)$ and (1,1). Similarly for (α,β) to values (1,1), (1/9,1) and (0.1,0.9), for means to the Be distribution that are equal to 0.5,0.1 and 0.9 respectively.

Note that we ran the algorithms over a range of hyperparameter values (e.g., (η,ν)=(XX,YY),(xx,yy) and (xx1,xx2) and did not observe significant performance degradation

### 3.3. Joint Posterior Distribution

Assuming the parameters z and t are *a priori* mutually independent, the joint posterior of the parameter vector $\Delta =\left\{z,t\right\}$ and hyperparameters $\Phi =\{s^{2},\omega \}$ can be expressed as:

(11) $$\begin{array}{c} f(\Delta ,\Phi |y)\propto f(y|\Delta )f(\Delta |\Phi )f(\Phi ), \end{array}$$

where $f\left(\Delta |\Phi \right)=f\left(z|\omega \right)f\left(t|s^{2}\right)$ and $f\left(\Phi \right)=f\left(s^{2}\right)f\left(\omega \right)$. The joint posterior distribution reduces to:

(12) $$\begin{array}{cc} p(\Delta ,\Phi |y)\;\propto \;\; & F\left(AZt\right)\times \prod_{g=1}^{G}\left(\left[\omega^{z_{g}}{\left(1-\omega \right)}^{\left(1-z_{g}\right)}\right]\times \prod_{i=1}^{Kg}N_{R^{+}}\left(t_{i};0,s^{2}\right)\right)\\ & \times Be\left(\omega ;\alpha ,\beta \right)\times IG\left(s^{2};\eta ,\nu \right), \end{array}$$

where $Z=\mathrm{diag}(\left[z_{1}1_{K_{1}},z_{2}1_{K_{2}},\cdots ,z_{g}1_{K_{g}}\right])$. The next paragraph describes the sampling strategy adopted to estimate the unknown parameter vector Δ and the hyperparameters Φ.

## 4. Bayesian Inference

To overcome the challenging derivation of Bayesian estimators associated with f(Δ,Φ|y), we use an efficient Markov chain Monte Carlo (MCMC) method to generate samples asymptotically distributed according to Equation (12). More precisely, we consider a variable-splitting inspired MCMC algorithm, namely the split-and-augmented Gibbs sampler (SPA, presented in the next paragraph) that was recently proposed in [18]. For SPA, we assume that Υ={b,c}, where b≈Ac and c≈Zt. This splitting decouples locally each vector of variables from the rest of the variables, which will in turn make the inference easier. The joint posterior distribution in Equation (11) can be extended as:

(13) $$\begin{array}{cc} p(\Delta ,\Upsilon ,\Lambda ,\Phi |y)\propto \; & F\left(AZt\right)\times \prod_{g=1}^{G}f\left(z_{g},t_{g}\right)\times \exp\left(-\frac{{\left\|Ac-(b-o_{1})\right\|}_{2}^{2}}{2\rho^{2}}\right)\\ & \times \exp\left(-\frac{{\left\|Zt-(c-o_{2})\right\|}_{2}^{2}}{2\rho^{2}}\right)\times Be\left(\omega ;\alpha ,\beta \right)\times IG\left(s^{2};\eta ,\nu \right)\\ & \times \exp\left(-\frac{{\left\|o_{1}\right\|}_{2}^{2}}{2\alpha^{2}}\right)\times \exp\left(-\frac{{\left\|o_{2}\right\|}_{2}^{2}}{2\alpha^{2}}\right), \end{array}$$

where $f\left(z_{g},t_{g}\right)=\left(\left[\omega^{z_{g}}{\left(1-\omega \right)}^{\left(1-z_{g}\right)}\right]\times \prod_{i=1}^{Kg}N_{R^{+}}\left(t_{i};0,s^{2}\right)\right)$ and the new terms are divergence functions such that the joint extended posterior distribution defines a proper probability distribution. The auxiliary variables $\Lambda =\{o_{1},o_{2}\}$ associated with the splitting variables Υ={b,c}, which allow to decrease the correlation between the variables by giving an additional degree of freedom to each of the former variables (see [18] for more details). Moreover, ρ,α>0 are user defined parameters. In Equation (13), it is possible to use a block Gibbs sampler (with proximal MCMC step in the Poisson noise case) to sample according to the conditional distributions of each of the unknown model parameters. In practice, strong correlations appear between z and t. Moreover, as z can be sparse, sampling $f(t,s^{2}|y,\Delta_{\setminus t},\Phi_{\setminus (s^{2})})$, where $H_{\setminus u}$ denotes the parameter vector H whose parameter u is omitted, via Gibbs sampling results in very slow convergence. Hence, we use a partially collapsed Gibbs sampler (PCGS) which provides better mixing and convergence properties [28,29,30,31,32,33]. The PCGS used here samples groups of variables (e.g., (z,t)) from their joint posterior distribution rather than from their conditional distributions. Sampling from the joint distribution is achieved by first marginalising some variables which are then sampled from their full conditional distribution [34,35]. Precisely, we sample sequentially the elements of Δ,Υ,Λ and Φ using moves that are summarised in Algorithm 1.
**Algorithm 1** Split and augmented—partially collapsed Gibbs sampling algorithm for activity estimation in PGET—version I.1:**Fixed input parameters**: Number of burn-in iterations $N_{\mathrm{bi}}$, total number of iterations $N_{\mathrm{MC}}$2:**Initialization** (k=0)• Set $z^{\left(0\right)},t^{\left(0\right)},b^{\left(0\right)},c^{\left(0\right)}$, $\omega^{\left(0\right)},{s^{2}}^{\left(0\right)}$$,o_{1}^{\left(0\right)},o_{2}^{\left(0\right)}$3:**Repeat ($1\leq k\leq N_{\mathrm{MC}}$)**1.Sample $\left({s^{2}}^{\left(k\right)},{t_{z_{g}=0}}^{\left(k\right)}\right)|\left(y,\omega^{\left(k-1\right)},\Delta_{\setminus t_{z_{g}=0}}^{\left(k-1\right)},\Upsilon^{\left(k-1\right)},\Lambda^{\left(k-1\right)}\right)$2.Sample $\left(z^{\left(k\right)},t^{\left(k\right)}\right)|\left(y,{s^{2}}^{\left(k\right)},\Upsilon^{\left(k-1\right)},\Lambda^{\left(k-1\right)}\right)$3.Sample $\omega^{\left(k\right)}|\left(y,{s^{2}}^{\left(k\right)},\Delta^{\left(k\right)},\Upsilon^{\left(k-1\right)},\Lambda^{\left(k-1\right)}\right)$4.Sample $b^{\left(k\right)}|\left(y,\Phi^{\left(k\right)},\Delta^{\left(k\right)},c^{\left(k-1\right)},\Lambda^{\left(k-1\right)}\right)$5.Sample $c^{\left(k\right)}|\left(y,\Phi^{\left(k\right)},\Delta^{\left(k\right)},b^{\left(k\right)},\Lambda^{\left(k-1\right)}\right)$6.Sample $o_{1}^{\left(k\right)}|\left(y,\Phi^{\left(k\right)},\Delta^{\left(k\right)},\Upsilon^{\left(k\right)},o_{2}^{\left(k-1\right)}\right)$7.Sample $o_{2}^{\left(k\right)}|\left(y,\Phi^{\left(k\right)},\Delta^{\left(k\right)},\Upsilon^{\left(k\right)},o_{1}^{\left(k\right)}\right)$4:**Set**k=k+1.

In Algorithm 1, $t_{z_{g}=0}$ denotes the elements of t whose corresponding labels in z are null. Similarly, $t_{z_{g}=1}$ denotes the elements of t whose labels are equal to 1. We introduce the details of sampling each step of the algorithm above in the Appendix A.

The algorithm is stopped after $N_{\mathrm{MC}}$ iterations, including the $N_{\mathrm{bi}}$ burn-in iterations, which correspond to the transient period of the sampler (determined visually from preliminary runs). The first $N_{\mathrm{bi}}$ samples are discarded and the remaining samples are used to approximate the following coupled Bayesian estimators that are particularly suitable for pin activity estimation problems. For the support of the activity vector or “presence maps”, which provides the probability of presence of pins, we use the marginal maximum a posteriori (MMAP) estimator [36,37]

(14) $$\begin{array}{c} z_{g}^{\mathrm{MMAP}}=\arg\max_{z_{g}\in \left\{0,1\right\}}f\left(z_{g}|y,\Delta_{\setminus z_{g}},\Phi ,\Upsilon ,\Lambda \right). \end{array}$$

As we are interested in estimating the pin intensities $x_{g}=z_{g}t_{g}=z_{g}[t_{1,g}\cdots ,t_{i,g},$ $\cdots ,t_{K_{g},g}]$, the empirical averages of the generated samples (conditional minimum mean squared error “CMMSE” estimates) of each pin pixel $x_{i,g}=z_{g}t_{i,g}$, conditionally on the estimated supports, is given by:

(15) $$\begin{array}{c} x_{i,g}^{\mathrm{CMMSE}}=E\left[x_{i,g}|z_{g}^{\mathrm{MMAP}},y,\Phi ,\Upsilon ,\Lambda \right], \end{array}$$

where if $z_{g}^{\mathrm{MMAP}}=0$, $x_{i,g}^{\mathrm{CMMSE}}=0$, otherwise $x_{i,g}^{\mathrm{CMMSE}}$ is given by:

(16) $$\begin{array}{c} x_{i,g}^{\mathrm{CMMSE}}=\frac{1}{\sum_{k>N_{\mathrm{bi}}}z_{g}^{\left(k\right)}}\sum_{k>N_{\mathrm{bi}}}^{}{\left(z_{g}t_{i,g}\right)}^{\left(k\right)}, \end{array}$$

The variance of active pin pixels can be computed by:

(17) $$\begin{array}{c} v_{i,g}^{2}=\frac{1}{\sum_{k>N_{\mathrm{bi}}}z_{g}^{\left(k\right)}}\sum_{k>N_{\mathrm{bi}}}^{}{\left({\left(z_{g}t_{i,g}\right)}^{\left(k\right)}-x_{i,g}^{\mathrm{CMMSE}}\right)}^{2}, \end{array}$$

Moreover, the average of pin pixel intensities can be computed as:

(18) $$\begin{array}{c} \hat{x_{g}}=\frac{1}{K_{g}}\sum_{i=1}^{K_{g}}x_{i,g}^{\mathrm{CMMSE}}, \end{array}$$

which can then assigned for the pixels forming each pin. The variance can be computed in same manner as in Equation (17).

## 5. Simulations Using Synthetic Datasets

In this section, we assess the performance of the proposed algorithm for cases where the data, given x, are generated according to the model used to perform Bayesian inference. More precisely, we define the linear operator A, compute Ax, and generate sinograms corrupted by additive idd Gaussian noise and by Poisson noise. We then use the corresponding algorithm to infer x. Cases where y is generated using the more realistic simulator will be discussed later in Section 6.

### 5.1. Data Creation

**Figure 2:**
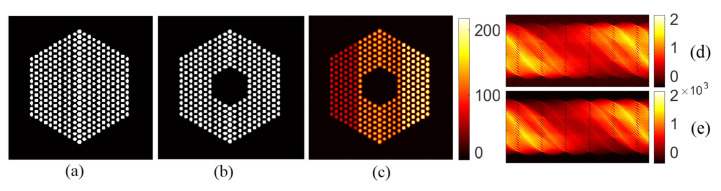
Simulated dataset. (**a**) The admissible support, (**b**) original binary mask, (**c**) ground truth activity profile when the peak value is set to 220, (**d**) sinogram generated with an iid Gaussian noise model with peak value = 220 and noise variance $\sigma^{2}=2\times 10^{-3}$, and (**e**) sinogram generated with a Poisson noise model with peak value = 220.

To assess the performance of the proposed approaches, we created a simulated activity profile akin to those expected in actual assemblies. This image is of size (182×182) and contains 331 pins spanning a total number of 5329 pixels. The image, depicted in Figure 2c presents three different pin activity levels, and 10% of pins are missing at the center of the assembly. The response matrix of the system $A\in R^{65,520\times 5329}$ was constructed by simulating the detector array response to a source of unit activity inside each pixel, using the ground truth presence maps depicted in Figure 2b. Here, the admissible support in Figure 2a contains all the possible pin locations in the assembly. This choice was made to confirm whether the algorithms are prone to underestimating of overestimating the number of pins actually present. The forward model in Equation (1), with the different noise models in Equations (2) and (3), is then used to generate the sinograms with the system response matrix A. Different activity peak values in {220,160,100,40,10} (accounting for different signal to noise ratios), are tested for the Poisson noise case. For the Gaussian noise case, the activity peak value was set to 220, and the noise variance in the Gaussian case is assumed independent and identically distributed idd different noise variances, belonging to $\left\{1,2,4,6,8\right\}\times 10^{-3}$, were considered. Note that the linear operator in the Poisson noise case is scaled to $A=A\times 10^{3}$ to keep the same activity profile as of the Gaussian noise case. Figure 2d,e show examples of the corresponding sinograms generated using the Poisson and the Gaussian noise models.

### 5.2. Quantitative Analysis

**Figure 3:**
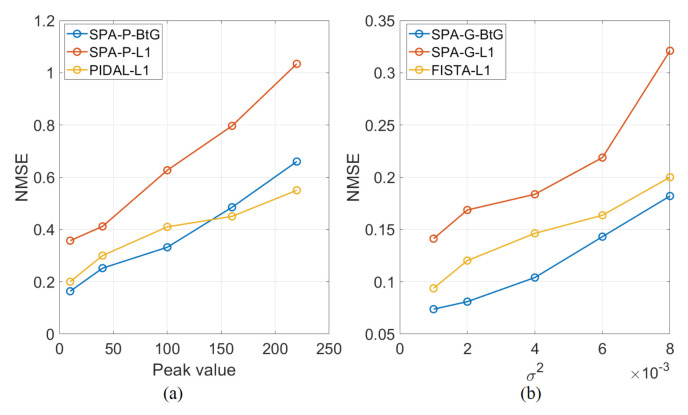
Normalized mean squared error (NMSE) plots for the (**a**) Poisson noise model with different peak values, (**b**) the Gaussian noise model when the maximum intensity is set to 220 and different noise variances.

The proposed approach is run on the simulated datasets described above. In all cases, a Markov chain of length $N_{\mathrm{bi}}=1.2\times 10^{4}$ and a burn-in period of length $N_{\mathrm{MC}}=4\times 10^{3}$ are used. The hyperparameters (ρ,α) of the split-and-augmented Gibbs sampler are set to (ρ,α)=(10,1) and those of the P-MYULA are set to $\left(\lambda ,\gamma \right)=\left(\rho^{2},\rho^{2}/4\right)$, as in [17]. The quantitative measure used to assess the quality of estimated activity profiles is the normalized mean square error (NMSE), defined as

(19) $$\begin{array}{c} \mathrm{NMSE}=\frac{{\left\|x-\hat{x}\right\|}_{2}^{2}}{{\left\|x\right\|}_{2}^{2}}, \end{array}$$

where $\hat{x}$ is an estimate of x. The proposed approach is compared against four of the existing approaches described earlier, namely, SPA-P-$\ell_{1}$ and PIDAL-$\ell_{1}$, for the Poisson noise model, and SPA-G-$\ell_{1}$ and FISTA-$\ell_{1}$ for the Gaussian noise model. For both SPA-P-$\ell_{1}$ and SPA-G-$\ell_{1}$, the Markov chain length $N_{\mathrm{MC}}$, burn-in period $N_{\mathrm{bi}}$, the hyperparameters (ρ,α) of the split-and-augmented Gibbs sampler, and those of the P-MYULA (λ,γ) are set as in the proposed approach. For the four methods, several values for the $\ell_{1}$ regularization parameter β are tested, from which we pick the one providing the lowest NMSE. Figure 3 shows plots of NMSE versus different activity profile peak values for the Poisson noise case, and different noise variances for a fixed activity profile peak value for the Gaussian noise case. We can observe that SPA-$\ell_{1}$ provides the highest NMSE, whereas SPA-BtG and FISTA/PIDAL-$\ell_{1}$ provide close results. This figure shows that, when comparing MMSE estimates, the proposed Bernoulli-based prior enforcing group sparsity is more efficient than the Exponential-based prior, for both noise models. Although MAP estimation based on the Exponential-based prior is appealing, the corresponding algorithms do not allow directly uncertainty quantification.

### 5.3. Qualitative Analysis

#### 5.3.1. The Proposed Method

**Figure 4:**
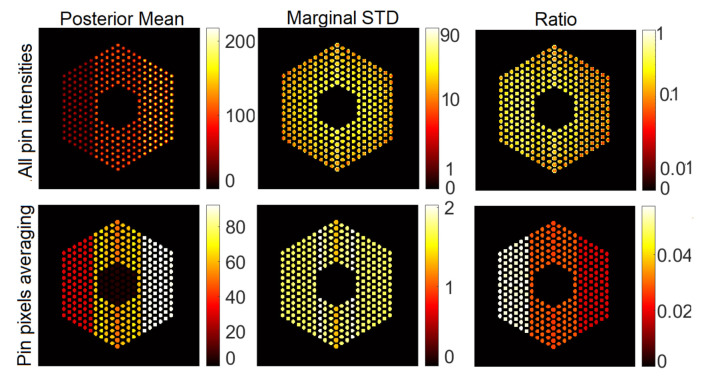
Results of the proposed approach using the Gaussian noise model, when the peak value is set to 220 and noise variance is set to $\sigma^{2}=2\times 10^{-3}$. Column 1: posterior mean, Column 2: marginal standard deviation and Column 3: ratio of marginal standard deviation divided by posterior mean. Row 1: All pixels, and Row 2: pin pixels averaging.

**Figure 5:**
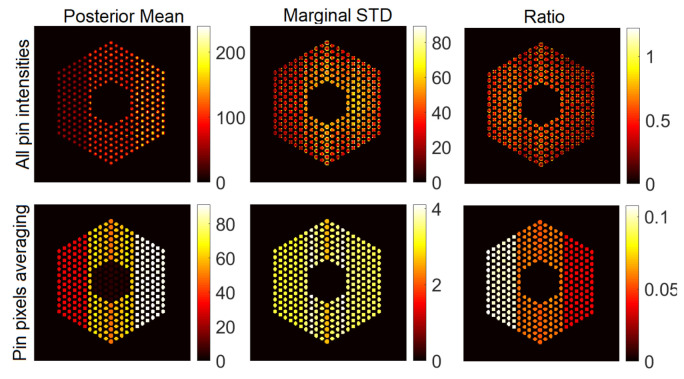
Results of the proposed approach using the Poisson noise model when the peak value is set to 220. Column 1: posterior mean; column 2: marginal standard deviation; and column 3: ratio of marginal standard deviation divided by posterior mean. Row 1: all pixels; row 2: pin pixels averaging.

In this section, we present visual results for activity estimation using the proposed approaches. For each case, we plot both the individual pin pixel activities and the average activity of pixels spanning each pin. Figure 4 and Figure 5 show examples of results for pin activity estimation and uncertainty quantification for the Gaussian and Poisson noise models, respectively. The peak value was set to 220 in both, and the noise variance of the Gaussian noise case was set to $\sigma^{2}=2\times 10^{-3}$. We can observe that the algorithm could successfully reconstruct the activity profile with both noise models. Moreover, it can be seen that the differences in pin activity levels are clearer when considering the average of pin pixel intensities as an activity estimate, and the uncertainty is much lower, in comparison with considering all pixel intensities. This is because averaging naturally contributes to reducing the noise in the activity estimates, equivalently enhancing the local signal to noise ratio. Consequently, the ratio of marginal standard deviation divided by the posterior mean shows clearer activity level differences. Hence, for the rest of the experiments in this paper, we will consider pin pixel averages as activity estimates.

**Figure 6:**
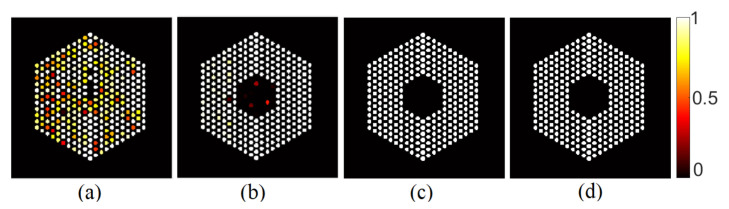
Probability of pin presence for (**a**) the Poisson noise case when peak value is set to 10, (**b**) the Gaussian noise case for peak value of 220 and noise variance $\sigma^{2}=1\times 10^{-2}$, and (**c**) the Poisson noise case when peak value is set to 220, and (**d**) the Gaussian noise case when the peak value is set to 220 and the noise variance is set to $\sigma^{2}=2\times 10^{-3}$.

Figure 6 depicts examples of estimated probabilities of pin presence in both the Poisson noise case, using different activity peak values, and the Gaussian noise case for two levels of signal quality. We can observe that for both the Gaussian and Poisson noise cases, when the peak value is reasonably high, the algorithm could identify with high confidence levels the 10% missing pins near the center of the assembly. However, when the peak value in the Poisson noise case decreases, and the noise variance in the Gaussian noise case increases, it becomes more difficult to identify the assembly configuration. In Figure 6a for instance, the algorithm tends to overestimate the number of pins present while pins in the outer ring of the assembly seem more difficult to identify with high confidence. While the comparison between Poisson and iid Gaussian noise is difficult as in the Poisson case the signal-to-noise ratio is pixel-dependent, we observed that for similar average SNR, the activity estimation is more challenging in the Poisson case, probably because of the non-stationary SNR.

#### 5.3.2. Comparison with Existing Methods

**Figure 7:**
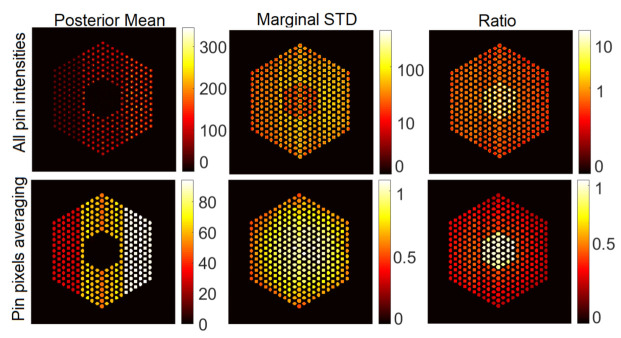
Pin activity estimation using SPA-P-$\ell_{1}$, using the Poisson noise model when peak value is set to 220. The results using the Gaussian noise model (SPA-G-$\ell_{1}$) using peak value = 220 and noise variance $\sigma^{2}=2\times 10^{-3}$ present similar trends and hence are not presented here.

**Figure 8:**
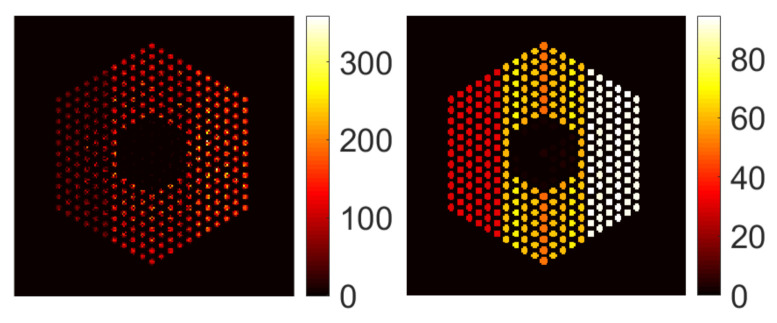
Pin activity estimation using PIDAL-$\ell_{1}$, using the Poisson noise model when the peak value is set to 220. (**Left**): all pin pixels; (**right**): average of pin pixels.

For completeness, Figure 7 shows examples of results with the SPA-P-$\ell_{1}$ approach for pin activity estimation using the Poisson noise model. The results for the Gaussian noise model (using SPA-G-$\ell_{1}$) present similar trends and hence are not included here. For all pin pixels and pin pixels averaging results, we can observe that the posterior means look similar to those of the proposed approach. However, from the ratio of marginal standard deviation divided by posterior mean, the SPA-P-$\ell_{1}$ approach is not confident about the 10% missing pins in the center. On the other hand, Figure 8 shows the results of the PIDAL-$\ell_{1}$ approach for pin activity estimation using Poisson noise model. Those of the Gaussian noise model using FISTA-$\ell_{1}$ are similar and hence are not presented here. We can observe that the maximum *a posteriori* estimate is similar to the MMSE using both the proposed approach and SPA-P-$\ell_{1}$. However, these MAP-based methods cannot provide uncertainty measures about the estimates.

## 6. Simulations Using Realistic Datasets

**Figure 9:**
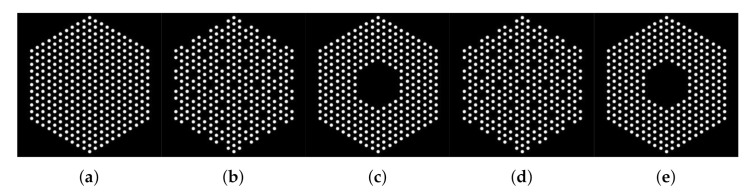
Actual binary masks of the simulated fuel assemblies. (**a**) Case “1”, (**b**) Case “2”, (**c**) Case “3”, (**d**) Case “4”, (**e**) Case “5”. In Case “1”, the mock-up fuel assembly hosted 331 ${}^{60}$Co pins of 8.879 g/cm${}^{3}$ density, which emitted 1.17 and 1.33 MeV gamma rays. In Cases “2” and “3”, 10% ${}^{60}$Co pins pins were removed at the center or uniformly. In Cases “4” and “5”, 10% ${}^{60}$Co pins pins were replaced by depleted uranium pins of the same size but higher density (10.4 g/cm${}^{3}$) and no activity.

**Figure 10:**
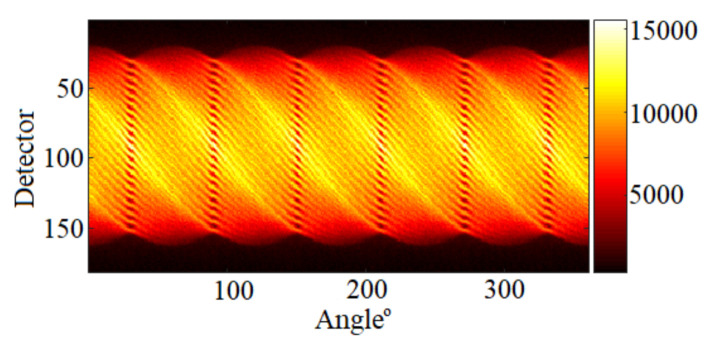
Simulated sinogram of Case “1”. The colour scale represents the detected photon counts.

In this section, we simulated the measurement of five fuel pin configurations in a mock-up water-water energetic reactor (WWER) fuel assembly using the software MCNP 6.2 [38], as shown in Figure 9. In this figure, we show the ground truth assembly configuration of each case. As in Case “1”, the mock-up fuel assembly hosted 331 ${}^{60}\mathrm{Co}$ pins, which emitted 1.17 and 1.33 MeV gamma rays. We simulated ${}^{60}\mathrm{Co}$ pins because they are used for the testing of the PGET system. In Cases “2” and “3”, we mimicked the scenario where fuel pins were missing by removing 10% fuel pins in the assembly. Each pin in the three cases is formed by a number of pixels belonging to {10–16}. In Cases “4” and “5”, 10% fuel pins were replaced by depleted uranium pins of the same size as the ${}^{60}\mathrm{Co}$ ones. These pins are not emitting measurable gamma rays. In the five cases, the activity in each white pixel is constant. In contrast to Section 5 where we used a linear operator A constructed using physical model of collimator-detector system response and simulated data using the linear model in Equation (1), here the data are generated using the MCNP 6.2 software that simulates all photon interactions in the PGET system [38]. More details on the simulation set up can be found in [22]. The simulated detection unit is a high-fidelity model of the PGET and consists of two collimated CdTe detector arrays on the top and bottom sides of the fuel assembly. The detector arrays rotate and scan the fuel bundle generating a 182×360 sinogram, as shown in Figure 10. Due to the mismatch between the observations and Ax, the robustness of the linear model is assessed using the two noise models mentioned earlier; the idd Gaussian and the Poisson noise models.

Different response matrices, namely the IDEAL, the FULL and the EMPIRICAL, were tested to estimate the pin activity. To calculate a response matrix A, pin locations are needed as input parameters to identify the pixels inside the pins and account for scattering and attenuation effects. To construct the IDEAL response matrices, the exact pin positions in each case (ground truth) are used. With such response matrices, we run the proposed algorithms to estimate the pin activity levels and to confirm whether the methods tend to underestimate the number of pins. For Case “1”, where no pins are missing, $A\in R^{65,520\times 5329}$. However, for the rest of the cases (Cases “2” to “5”) where 10% of the pins are missing, the response matrix of the system is of size $A\in R^{65,520\times 4732}$. In practice, these IDEAL response matrices are not available as we aim precisely at identify which pins are present. A more realistic scenario consists of assuming that all the pins are *a priori* present (although they may not). This assumption is accurate in Case 1, but it expected to introduce errors for the other cases, in particular if a large number of pins is missing. The resulting matrix is of size 65,520×5329 and it coincides with the IDEAL matrix of Case 1. The last strategy considered here to build A is referred to as EMPIRICAL and consists of identifying, as a pre-processing step, the likely pin support using a fast inversion algorithm (filtered backprojection (FBP), as in [22]). FBP provides a coarse activity map which is then thresholded to identify potential pin locations. This approach is more evolved than using the FULL A and can discard correctly potential pin locations, but it can also remove legit pin locations if the threshold is set incorrectly. Using the empirical matrices A, a matrix is created for each of the five cases.

### 6.1. The IDEAL Response Matrix

#### 6.1.1. Results Using the Proposed Approach

**Figure 11:**
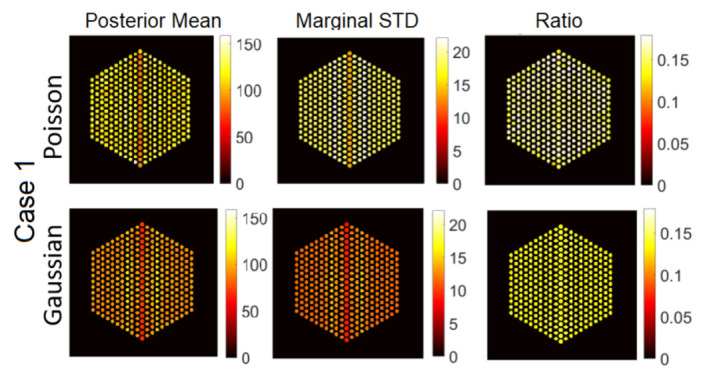
The IDEAL response matrix: Pin pixels averaging of the proposed approach for Case “1”. (**Left-hand column**): posterior mean, (**middle column**): marginal standard deviation, and (**right-hand column**): the ratio of marginal standard deviation divided by posterior mean.

**Figure 12:**
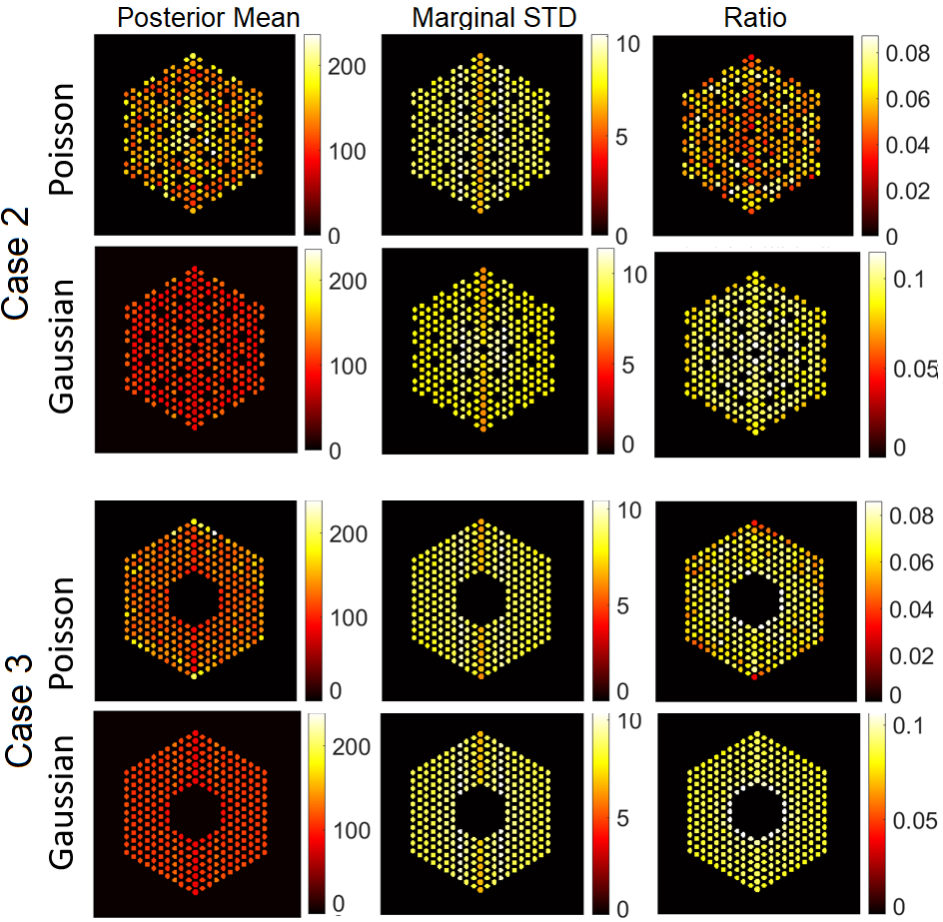
The IDEAL response matrix: Pin pixels averaging of the proposed approach for Case “2” and Case “3”. (**Left-hand column**): posterior mean, (**middle column**): marginal standard deviation, and (**right-hand column**): the ratio of marginal standard deviation divided by posterior mean.

**Figure 13:**
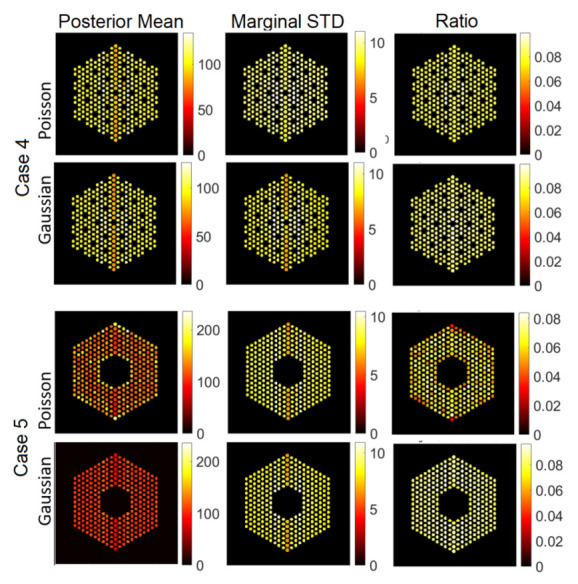
The IDEAL response matrix: Pin pixels averaging of the proposed approach for Case “4” and Case “5”. (**Left-hand column**): posterior mean, (**middle column**): marginal standard deviation, and (**right-hand column**): the ratio of marginal standard deviation divided by posterior mean.

**Figure 14:**
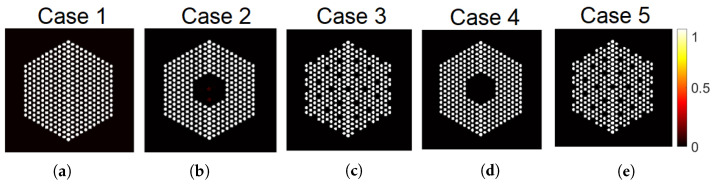
The IDEAL response matrix: Probability of presence of pins using the proposed approach for the Poisson noise model. (**a**) Case “1”; (**b**) Case “2”, (**c**) Case “3”, (**d**) Case “4” and (**e**) Case “5”. The Gaussian noise model showed similar trends and hence not presented.

In this section, we test the performance of the proposed approach using the IDEAL response matrices. In this context, the linear model in Equation (1) is expected to be a good approximation of the actual forward model. In all cases, the hyperparameters of the model are set like those described in Section 5. Figure 11, Figure 12 and Figure 13 show the results of activity estimation and the associated uncertainty measures, for Case 1 (full assembly), Cases 2 and 3 (missing pins) and Cases 4 and 5 (replaced pins), respectively. In these figures, we can observe that the Gaussian noise model generally provides more uniform pin activities compared to when using the Poisson noise model. Moreover, the activity profiles using the Gaussian noise model are lower, compared to the Poisson noise model, as it fitted the data better and hence improved the convergence and mixing properties of the MCMC chains. Figure 14 shows the posterior probabilities of presence of pins in the five investigated assembly patterns, using the Poisson noise model. We can observe that existing pins have a probability of presence of almost 1, thus the proposed approach is very confident in assessing the presence of pins. Similar results have been obtained using the Gaussian model and are thus not presented here. Both noise models allow correct pin identification for the five assembly patterns tested, as no additional pins are identified as missing.

#### 6.1.2. Comparison with Existing Methods

**Figure 15:**
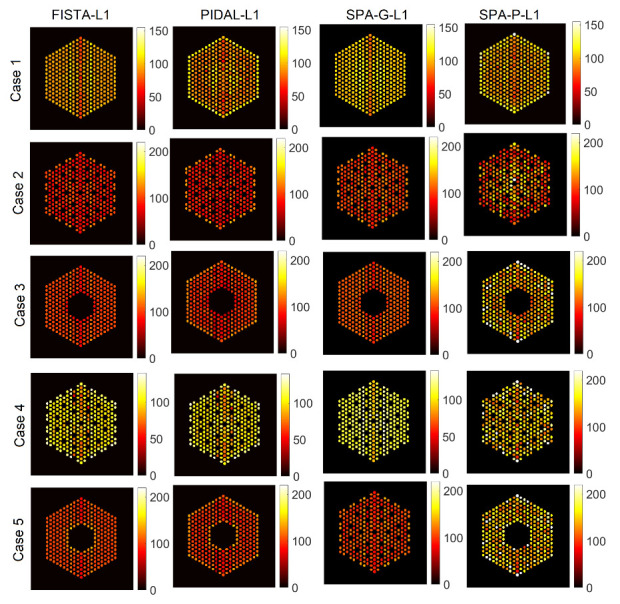
The IDEAL response matrix: Activity estimation using pin pixels averaging using existing methods. The regularization parameter β was set to $1\times 10^{-5}$ in the four methods.

In this part, we compare the proposed approach against the four existing methods described earlier that are SPA-G-$\ell_{1}$ and FISTA-$\ell_{1}$ for the Gaussian noise case, and SPA-P-$\ell_{1}$ and PIDAL-$\ell_{1}$ for the Poisson noise case. Figure 15 shows the activity estimation results of the five assembly patterns using these methods. We can observe that the results are similar to those obtained by the proposed approach. Moreover, all of the methods correctly identify the assembly pattern of all datasets, as in addition to the pins already identified as missing, no extra pins are. It is also worth mentioning here that the convergence of PIDAL-$\ell_{1}$, which considers a Poisson likelihood, was much slower than FISTA-ℓ1 which considers a Gaussian likelihood. On the other hand, in Table 1, we show the number of particles (NPS) for the proposed approaches and the four existing methods. NPS is the mean of active pin pixels in each case. For each case, we estimated the activity of each pin by summing up pin pixel intensities and created a histogram of pin activities. The mean of this histogram is then computed, which represents the average fuel activity of each case. We can observe that, in general, the methods considering a Gaussian likelihood (FISTA-$\ell_{1}$, SPA-G-BtG and SPA-G-$\ell_{1}$) provide fairly close results to the ground truth NPS.

Table 2 provides the average computation time of the proposed approach and the existing methods. The algorithms are implemented in MATLAB and the experiments are carried out on a laptop with a 2.8 GHz processor CPU, with 16 GB of RAM, under Microsoft Windows 10. It is clear that FISTA-ℓ1 provides the lowest computation cost and PIDAL-ℓ1 provides the highest computation complexity which is because it requires more iterations to converge. Despite the relatively high computation time of the proposed algorithms, they do not require fine tuning of the regularization parameter as in PIDAL/FISTA-ℓ1, SPA-P-ℓ1 and SPA-G-ℓ1. Moreover, it can provide the probability of presence of pins and quantify their uncertainty measures in contrast to the other algorithms, as outlined in Table 2.

### 6.2. The FULL Response Matrix

#### 6.2.1. Results Using the Proposed Approach

**Figure 16:**
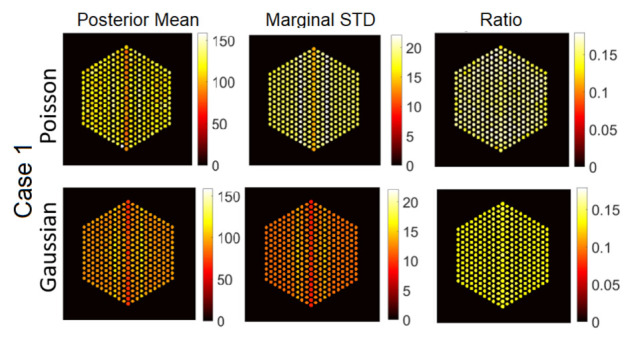
The FULL response matrix: pin pixels, averaging of the proposed approach for Case 1 “Full assembly”. (**Left-hand column**): posterior mean; (**middle column**): marginal standard deviation; and (**right-hand column**): the ratio of marginal standard deviation divided by posterior mean.

**Figure 17:**
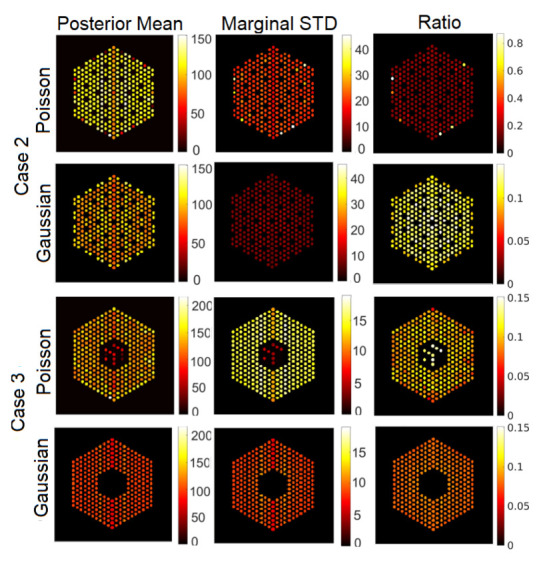
The FULL response matrix: pin pixels averaging of the proposed approach for Case 2 “Uniform Missing Assembly” and Case 3 “Center Missing Assembly”. (**Left-hand column**): posterior mean; (**middle column**): marginal standard deviation (in log-scale); and (**right-hand column**): the ratio of marginal standard deviation divided by posterior mean.

**Figure 18:**
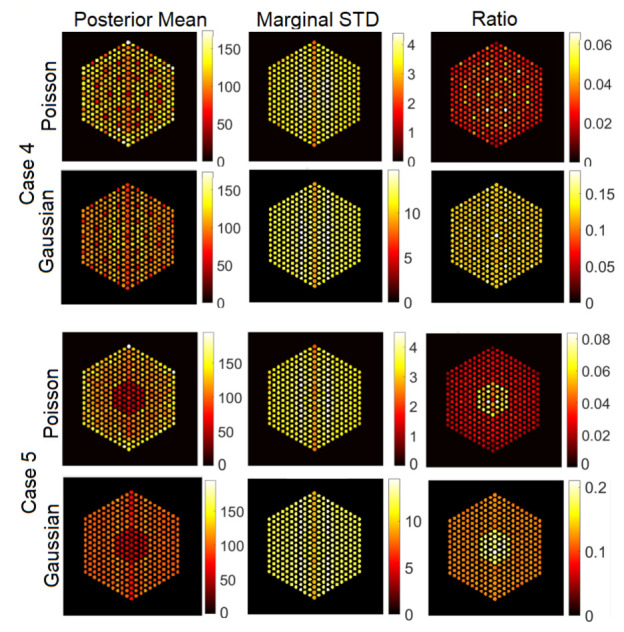
The FULL response matrix: pin pixels averaging of the proposed approach for Case “4” and Case “5”. (**Left-hand column**): posterior mean; (**middle column**): marginal standard deviation; and (**right-hand column**): the ratio of marginal standard deviation divided by posterior mean.

**Figure 19:**
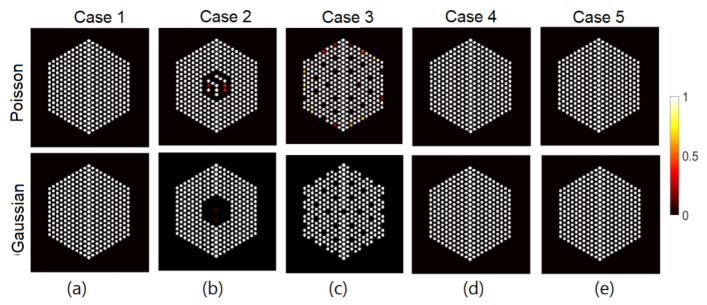
The FULL response matrix: probability of presence of pins using the proposed approach. (**a**) Case “1”; (**b**) Case “2”, (**c**) Case “3”, (**d**) Case “4”, and (**e**) Case “5”.

The IDEAL response matrices deployed in the previous subsection are not available, as we aim precisely at identify which pins are present. In this subsection, we investigate a more realistic scenario, consisting of the assumption that all the pins are *a priori* present (although, they may not be). This assumption is accurate in Case 1, but it expected to introduce errors for the other cases, as we will see below. The resulting matrix is of size $A\in R^{65,520\times 5329}$. The hyperparameters of the proposed methods are set to same values as in the experiments in the Section 6.1. Figure 16, Figure 17 and Figure 18 show the results activity estimation results obtained by our methods for the five cases. We can see that the algorithms could fairly accurately identify the assembly pattern of cases “1”, “2” and “3” but not for cases “4” and “5”. This can also be observed in the uncertainty maps of these cases. Moreover, the Gaussian noise model seems more robust in missing pins identification than using the Poisson noise model. On the other hand, Figure 19 shows the probability of presence of pins in each assembly. We can observe that the assembly patterns of cases “1”, “2”, and “3” are correctly identified, but not for cases “4” and “5”. In cases “4” and “5”, the cobalt pins are replaced by depleted UO2 and the Compton scattering cross-section of UO2 is larger than cobalt’s. UO2 pins are simulated as non-radioactive, as their radioactivity is negligible and UO2 could be used as a high-density fuel replacement material in diversion scenarios. However, UO2 pins are scattering centers of the gamma rays emitted by the surrounding fuel pins and the use of the FULL response matrix induces the algorithm to mis-classify UO2 pins as low-activity cobalt pins. One strategy to mitigate this effect could rely on narrowing the energy detection window to exclude the low-energy contribution from scattering reactions.

#### 6.2.2. Comparison with Existing Methods

**Figure 20:**
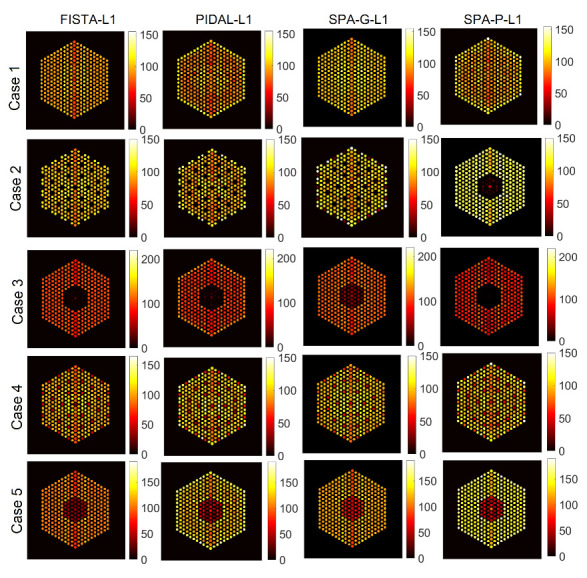
The FULL response matrix: activity estimation using pin pixels averaging using existing methods for the five cases. The regularization parameter β was set to $1\times 10^{-5}$ in the four methods.

As in the previous section, the proposed approach is compared against the four approaches described earlier. Figure 20 shows the activity estimation results of the five assembly configurations. We can observe that for the missing center case, there are still some low amplitude identified pins at the center. Moreover, the proposed approach provides better results, in addition to being able to quantify the uncertainty measures of the estimates and also localize the pins.

### 6.3. The EMPIRICAL Response Matrix

**Figure 21:**
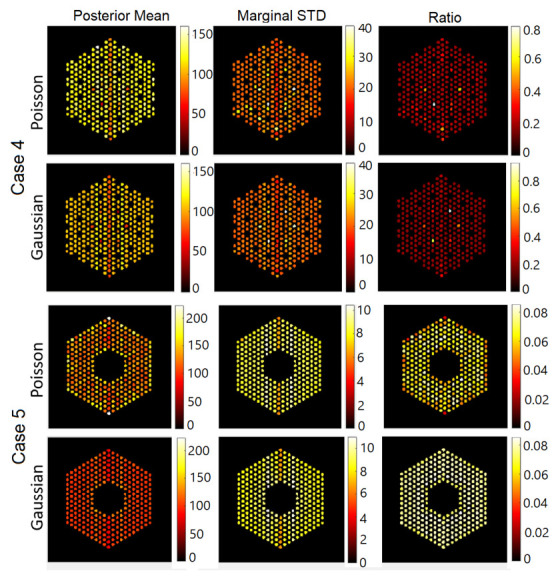
The EMPIRICAL response matrix: pin pixels averaging of the proposed approach for Case “4” and Case “5” using the “EMPIRICAL” response matrix. (**Left-hand column**): posterior mean; (**middle column**): marginal standard deviation; and (**right-hand column**): the ratio of marginal standard deviation divided by posterior mean.

**Figure 22:**
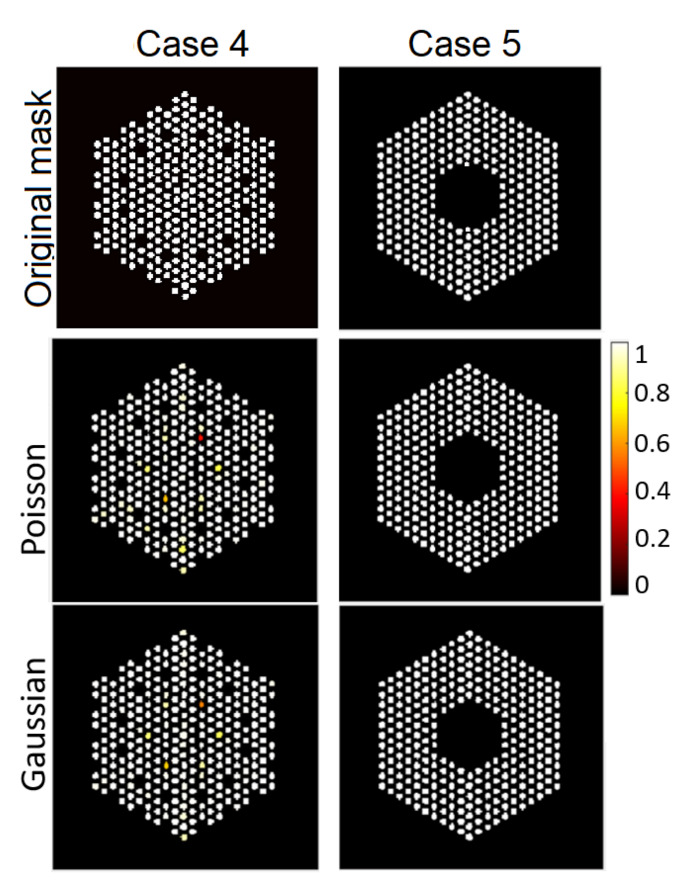
The EMPIRICAL response matrix: probability of presence of pins using the proposed approach. (**Left**): Case “4”; (**right**): Case “5”.

**Figure 23:**
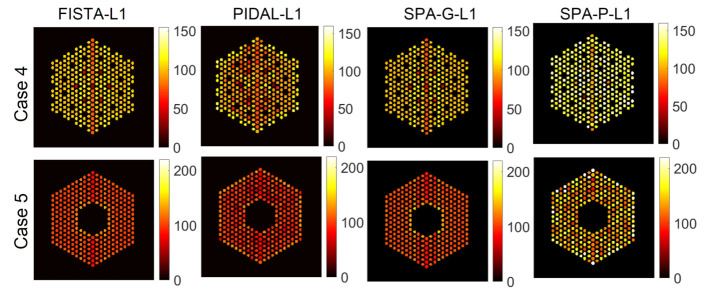
The EMPIRICAL response matrix: activity estimation using pin pixels averaging using existing methods, for Cases “4” and “5”. The regularization parameter β was set to $1\times 10^{-5}$ in the four methods.

As outlined in Section 6.2, the proposed approach, and existing methods, find it difficult to provide reliable estimates for cases “4” and “5”, which is probably because of the mismatch between the linear operator and the observations. Hence, the last strategy considered here is to build A by identifying as a pre-processing step, the likely pin support using a fast inversion algorithm (filtered backprojection (FBP), as in [22]). FBP provides a coarse activity map which is then thresholded to identify potential pin locations. This approach is more evolved than using the FULL A and can discard correctly potential pin locations. Using the empirical matrices A, a matrix is created for each of the last two cases (Case 4 and 5), which is of size $A\in R^{65,520\times 4899}$. Figure 21 and Figure 22 show pin activity estimation and presence maps using both the Poisson and Gaussian noise models. We can observe that the assembly patterns of both cases are correctly identified, although in the Case “4”, there are still some missing pixels identified as existing but with low probability of presence, as in Figure 22. On the other hand, Figure 23 shows a comparison with the existing approaches, which are similar to those obtained using the proposed approach but without associated uncertainty measures and probability of presence.

## 7. Conclusions and Future Work

This paper introduced a hierarchical Bayesian model for pin activity estimation in PGET images, using a linear forward model. Due to the linear approximation, the classical Poisson noise assumption is likely to not hold and we assessed the robustness of the model tested using two different noise models. Prior distributions were assigned to the unknown model parameters and Bayesian inference was performed using a split and augmented—partially collapsed Gibbs sampler. The proposed approaches can provide uncertainty measures to the estimates and are fully automatic in the sense that they can estimate the model associated hyperparameters. Different response matrices in the linear approximation were also investigated. We observed that the Gaussian noise model provided better results, as it seemed more robust to model mismatch. The proposed approaches can provide uncertainty measures and posterior probabilities of presence, which cannot be obtained using classical optimisation methods. However, this comes at a higher computation cost. Future work should investigate alternative approaches to model the forward model.

## Figures and Tables

**Table 1:** The IDEAL response matrix: Comparison of activity estimations ($\times 10^{7}$), represented by number of particle (NPS), for the proposed approaches and the four existing methods. NPS is the mean of active pin pixels in each case. Estimated activities close to the ground truth are highlighted in bold. Second close values are underlined.

	SPA-P-BtG	SPA-G-BtG	SPA-P-$\ell_{1}$	SPA-G-$\ell_{1}$	PIDAL-$\ell_{1}$	FISTA-$\ell_{1}$	Ground Truth
**Case 1**	2.00	1.49	2.244	1.65	**1.512**	1.524	1.51
**Case 2**	2.44	1.54	2.89	**1.72**	1.47	1.513	1.70
**Case 3**	2.10	1.58	2.37	**1.77**	1.49	1.624	1.70
**Case 4**	2.25	1.61	1.68	1.83	1.57	**1.68**	1.70
**Case 5**	2.16	1.65	2.47	1.80	**1.69**	**1.69**	1.70

**Table 2:** Computation time (in minutes), using CPU for the proposed approach and state of the art algorithms.

Method	SPA-P-BtG	SPA-G-BtG	SPA-P-$\ell_{1}$	SPA-G-$\ell_{1}$	PIDAL-$\ell_{1}$	FISTA-$\ell_{1}$
**Computation time (min)**	50	60	45	55	75	**3**
**Uncertainty maps**	Yes	Yes	Yes	Yes	No	No

## Data Availability

The data that support the findings of this study are available from the corresponding authors, A.K.E. and Y.A., upon reasonable request.

## Appendix A

We now detail each sampling step of Algorithm 1 as follows:

*Sampling $\left(s^{2},t_{z_{g}=0}\right)|\left(y,\Phi_{\setminus s^{2}},\Delta_{\setminus t_{z_{g}=0}},\Upsilon ,\Lambda \right)$*: We can notice that:

$$\begin{array}{c} f\left(s^{2},t_{z_{g}=0}|y,\Phi_{\setminus s^{2}},\Delta_{\setminus t_{z_{g}=0}},\Upsilon ,\Lambda \right)=f\left(s^{2}|y,\Phi_{\setminus s^{2}},\Delta_{\setminus t_{z_{g}=0}},\Upsilon ,\Lambda \right)f\left(t_{z_{g}=0}|y,\Phi ,\Delta_{\setminus t_{z_{g}=0}},\Upsilon ,\Lambda \right), \end{array}$$

where:

(A1) $$\begin{array}{c} f\left(s^{2}|y,\Phi_{\setminus s^{2}},\Delta_{\setminus t_{z_{g}=0}},\Upsilon ,\Lambda \right)=\int f\left(s^{2}|y,\Phi_{\setminus s^{2}},\Delta ,\Upsilon ,\Lambda \right)dt_{z_{g}=0}. \end{array}$$

Thus, sampling from $\left(s^{2},t_{z_{g}=0}\right)|\left(y,\Phi_{\setminus s^{2}},\Delta_{\setminus t_{z_{g}=0}},\Upsilon ,\Lambda \right)$ can be achieved by sampling sequentially $s^{2}$ from $f\left(s^{2}|y,\Phi_{\setminus s^{2}},\Delta_{\setminus t_{z_{g}=0}},\Upsilon ,\Lambda \right)$, and then $t_{z_{g}=0}$ from

$f\left(t_{z_{g}=0}|y,\Phi ,\Delta_{\setminus t_{z_{g}=0}},\Upsilon ,\Lambda \right)$. To compute $f\left(s^{2}|y,\Phi_{\setminus s^{2}},\Delta ,\Upsilon ,\Lambda \right)$ in Equation (A1), we keep only the parameters that depend on $s^{2}$ in the joint posterior distribution in Equation (11), this leads to:

(A2) $$\begin{array}{c} f\left(s^{2}|y,\Phi_{\setminus s^{2}},\Delta ,\Upsilon ,\Lambda \right)\propto \prod_{n=1}^{N}N_{R^{+}}\left(t_{n};0,s^{2}\right)\times IG\left(s^{2};\eta ,\nu \right). \end{array}$$

Due to the conjugacy of the distributions in Equation (A2), sampling from $f\left(s^{2}|y,\Phi_{\setminus s^{2}},\Delta ,\Upsilon ,\Lambda \right)$ can be achieved by sampling from the following inverse-gamma distribution [39]:

(A3) $$\begin{array}{c} s^{2}|\left(y,\Phi_{\setminus s^{2}},\Delta ,\Upsilon ,\Lambda \right)∼\mathrm{IG}\left(\eta +\frac{N}{2},\nu +\frac{\sum_{n=1}^{N}t_{n}^{2}}{2}\right). \end{array}$$

However, marginalizing $t_{z_{g}=0}$ out the conditional distribution in Equation (A3), according to Equation (A1), gives:

(A4) $$\begin{array}{c} s^{2}|\left(y,\Phi_{\setminus s^{2}},\Delta_{\setminus t_{z_{g}=0}},\Upsilon ,\Lambda \right)∼\mathrm{IG}\left(\eta +\frac{N_{1}}{2},\nu +\frac{\sum_{n\in I_{1}}t_{n}^{2}}{2}\right), \end{array}$$

where $N_{1}=\mathrm{card}\left(t_{z_{g}=1}\right)$ and $I_{1}=\left\{n|z_{n}=1\right\}$.

On the other hand, sampling $f\left(t_{z_{g}=0}|y,\Phi ,\Delta_{\setminus t_{z_{g}=0}},\Upsilon ,\Lambda \right)$ reduces to sampling its elements independently from their Gaussian prior distribution defined in Equation (6).

*Sampling (z,t)|(y,Φ,Υ,Λ)*: Updating (z,t) is achieved using the same marginalization trick as in Step (a) of Algorithm 1, i.e.:

$$\begin{array}{c} f\left(z,t|y,\Phi ,\Upsilon ,\Lambda \right)=f\left(z|y,\Phi ,\Upsilon ,\Lambda \right)f\left(t|y,\Delta_{\setminus t},\Phi ,\Upsilon ,\Lambda \right). \end{array}$$

It can be seen from Equation (11) that:

$$f\left(z|y,\Phi ,t,\Upsilon ,\Lambda \right)\propto$$

(A5) $$\begin{array}{c} \prod_{g=1}^{G}\left(\left[\omega^{z_{g}}{\left(1-\omega \right)}^{\left(1-z_{g}\right)}\right]\times {\left(2\pi \rho^{2}\right)}^{-\frac{K_{g}}{2}}\exp\left(-\frac{{\left\|z_{g}t_{g}-(c_{g}-{o_{2}}_{g}\right\|}_{2}^{2})}{2\rho^{2}}\right)\right), \end{array}$$

where $t_{g}\in R^{K_{g}}$ and that

(A6) $$\begin{array}{cc} \; & f\left(z_{g}=e|y,\Delta_{\setminus (z_{g^{\prime }},t_{g^{\prime }})},\Phi ,\Upsilon ,\Lambda \right)\propto {\bar{\omega }}_{g}^{\left(e\right)}\;\;\;\;\forall \left(g\right), \end{array}$$

where e∈{0,1} and

(A7) $$\begin{array}{ccc} \log({\bar{\omega }}_{g}^{\left(e\right)}) & = & -\frac{K_{g}}{2}\log\left(2\pi (\rho^{2}+es^{2})\right)\\ & - & \frac{{c_{g}}_{2}^{2}}{2(\rho^{2}+es^{2})}+\log\left(p(e|\omega )\right)+e\log\left[\prod_{i=1}^{K_{g}}\mathrm{erfc}\left(-\frac{s^{2}c_{i}^{2}}{\sqrt{2s^{2}\rho^{2}\left(\rho^{2}+s^{2}\right)}}\right)\right], \end{array}$$

where erfc(·) is the complementary error function (e.g., erfc(·)=1−erf(·)). Consequently, the label $z_{g}$ can be drawn from its conditional distribution (where $t_{g}$ has been marginalised) by drawing randomly from {0,1} with probabilities given by:

(A8) $$\begin{array}{cc} \; & f\left(z_{g}=e|y,\Delta_{\setminus (z_{g^{\prime }},t_{g^{\prime }})},\Phi ,\Upsilon ,\Lambda \right)=\frac{{\bar{\omega }}_{g}^{\left(e\right)}}{{\bar{\omega }}_{g}^{\left(0\right)}+{\bar{\omega }}_{g}^{\left(1\right)}}. \end{array}$$

Moreover, the elements of z can be updated in a parallel manner using the fact that $f\left(z|y,\Phi ,\Delta_{\setminus z},\Upsilon ,\Lambda \right)=\prod_{g}f\left(z_{g}|y,\Delta_{\setminus t},\Phi ,\Upsilon ,\Lambda \right)$.

Once z is sampled from (A8), sampling *$t|\left(y,\Delta_{\setminus t},\Phi ,\Upsilon ,\Lambda \right)$* can be achieved by sampling from the following multivariate Gaussian distribution:

$$\begin{array}{cc} f\left(t|y,\Delta_{\setminus t},\Phi ,\Upsilon ,\Lambda \right)\;\propto & \;\left(\prod_{n=1}^{N}N_{R^{+}}\left(t_{n};0,s^{2}\right)\right)\times \exp\left(-\frac{{\left\|Zt-(c-o_{2}\right\|}_{2}^{2})}{2\rho^{2}}\right) \end{array}$$

(A9) $$\begin{array}{c} t|\left(y,\Delta_{\setminus t},\Phi ,\Upsilon ,\Lambda \right)∼N_{R^{+}}\left(t;\mu_{t},\Sigma_{t}\right), \end{array}$$

where:

$$\begin{array}{c} \left\{\begin{array}{c} \mu_{t}=\left(\frac{Zc}{\rho^{2}}\right)\Sigma_{t},\\ \Sigma_{t}={\left(\mathrm{diag}\left(s^{-2}\right)+\rho^{-2}Z^{T}Z\right)}^{-1}. \end{array}\right. \end{array}$$

Note that $Z^{T}Z=Z$ as Z is a diagonal matrix. Hence, $\Sigma_{t}$ is a diagonal covariance matrix, which simplifies the sampling process.

*Sampling $\omega |(y,\Delta ,\Phi_{\setminus \omega },\Upsilon ,\Lambda )$*: Due to the conjugacy of the hierarchical prior model f(z|ω)f(ω), the full conditional distribution of ω reduces to the following beta distribution:

(A10) $$\begin{array}{c} \omega |\left(y,\Delta ,\Phi_{\setminus \omega },\Upsilon ,\Lambda \right)∼Be\left(\alpha +\sum_{g=1}^{G}K_{g}z_{g},\beta +N-\sum_{g=1}^{G}K_{g}z_{g}\right). \end{array}$$

*Sampling $b|\left(y,\Delta ,\Phi ,\Upsilon_{\setminus b},\Lambda \right)$*: It can be seen from Equation (13) that the full conditional distribution of b reduces to:

(A11) $$\begin{array}{c} f\left(b|y,b,c,o_{1}\right)\;\propto \;F\left(b\right)\times \exp\left(-\frac{{\left\|Ac-(b-o_{1})\right\|}_{2}^{2}}{2\rho^{2}}\right). \end{array}$$

When F(b)=P(b), due to the non-differentiability of this conditional distribution, we consider the proximal Moreau-Yoshida-unadjusted Langevin algorithm (P-MYULA) proposed in [20] to generate samples asymptotically distributed according to Equation (A11). Note also that [40] can be used which can provide faster convergence. The Markov chain of P-MYULA can be written as:

(A12) $$\begin{array}{c} b^{\left(k+1\right)}=\left(1-\frac{\gamma }{\lambda }\right)b^{\left(k\right)}-\gamma \nabla f\left(b^{\left(k\right)}\right)+\frac{\gamma }{\lambda }{\mathrm{prox}}_{g}^{\lambda }\left(b^{\left(k\right)}\right)+\sqrt{2\gamma }\;\;N_{R^{+}}\left(0,1\right), \end{array}$$

where

(A13) $$\begin{array}{ccc} f\left(b\right)=\frac{{\left\|Ac-(b-o_{1})\right\|}_{2}^{2}}{2\rho^{2}}, & \; & g(b)=-\log P(b),\\ \nabla f\left(b\right)=\frac{b-Ac-o_{1}}{\rho^{2}}, & \; & {\mathrm{prox}}_{g}^{\lambda }\left(b\right)=\frac{1}{2}\left(b-\lambda +\sqrt{{\left(b-\lambda \right)}^{2}+4\lambda y}\right). \end{array}$$

However, when $F\left(b\right)=f\left(y|b\right)={\left(2\pi \sigma^{2}\right)}^{-N/2}\exp\left(-\frac{1}{2\sigma^{2}}{\left\|y-b\right\|}_{2}^{2}\right)$, the full conditional distribution of b reduces to sampling from the following multivariate Gaussian distribution

(A14) $$\begin{array}{c} p\left(b|\Delta ,\Phi ,\Upsilon_{\setminus b},\Lambda \right)\;\propto N\left(b;\mu_{b},\Sigma_{b}\right), \end{array}$$

where:

(A15) $$\begin{array}{c} \mu_{b}=\left(\sigma^{-2}y+\rho^{-2}Ac\right)\Sigma_{b}^{-1},\;\mathrm{and}\;\;\;\;\Sigma_{b}=\frac{\sigma^{2}\rho^{2}}{\sigma^{2}+\rho^{2}}I_{N}. \end{array}$$

A simpler splitting strategy using only one split can be adopted when the noise model is Gaussian, that is h=Zt, thus eliminating one extra variable form the algorithm in Algorithm 1. We have tested the performance of the two splittings and did not find noticeable differences in the results. So, we keep the two splittings strategy described above for consistency with the Poisson noise model. Note that the noise variance $\sigma^{2}$ in the Gaussian noise case can be estimated using the method proposed in [41].

*Sampling $c|\left(\Delta ,\Phi ,\Upsilon_{\setminus c},\Lambda \right)$:* By cancelling out the terms that do not depend on c in Equation (13), the full conditional distribution of c can be written as:

(A16) $$\begin{array}{cc} p(c|\Delta ,\Phi ,\Upsilon_{\setminus c},\Lambda )\;\propto \;\; & \exp\left(-\frac{{\left\|Ac-(b-o_{1})\right\|}_{2}^{2}}{2\rho^{2}}\right)\times \exp\left(-\frac{{\left\|Zt-(c-o_{2}\right\|}_{2}^{2}}{2\rho^{2})}\right), \end{array}$$

which reduces to sampling from the following multivariate Gaussian distribution:

(A17) $$\begin{array}{c} p\left(c|\Delta ,\Phi ,\Upsilon_{\setminus c},\Lambda \right)\;=N\left(c;\mu_{c},\Sigma_{c}\right), \end{array}$$

where:

(A18) $$\begin{array}{c} \mu_{c}=\left(A^{T}\left(b-o_{1}\right)+Zt-o_{2}\right){\left(A^{T}A+I\right)}^{-1}\;\mathrm{and}\;\;\;\;\Sigma_{c}=\rho^{2}{\left(A^{T}A+I\right)}^{-1}. \end{array}$$

Sampling directly from (A17) can be computationally intensive since, e.g., it requires inversion of the precision matrix, sampling $c|\left(\Delta ,\Phi ,\Upsilon_{\setminus c},\Lambda \right)$ can be efficiently achieved by the exact perturbation-optimization (E-PO) algorithm proposed in [42].

*Sampling $o_{1}|\left(\Delta ,\Phi ,\Upsilon ,\Lambda_{\setminus o_{1}}\right)$*: It can be seen that the full conditional distribution of $o_{1}$ can be written as:

(A19) $$\begin{array}{ccc} \; & p\left(o_{1}|\Delta ,\Phi ,\Upsilon ,\Lambda_{\setminus o_{1}}\right)\;\propto \; & \exp\left(-\frac{{\|Ac-(b-o_{1})\|}_{2}^{2}}{2\rho^{2}}\right)\times \exp\left(-\frac{{\left\|o_{1}\right\|}_{2}^{2}}{2\alpha^{2}}\right), \end{array}$$

which reduces to sampling from the following multivariate Gaussian distribution:

(A20) $$\begin{array}{c} p\left(o_{1}|\Delta ,\Phi ,\Upsilon ,\Lambda_{\setminus o_{1}}\right)\;\propto N\left(o_{1};\mu_{o_{1}},\Sigma_{o_{1}}\right), \end{array}$$

where:

(A21) $$\begin{array}{c} \mu_{o_{1}}=\frac{\alpha^{2}}{\rho^{2}+\alpha^{2}}\left(Ac-b\right)\;\mathrm{and}\;\;\;\;\Sigma_{o_{1}}=\frac{\rho^{2}\alpha^{2}}{\rho^{2}+\alpha^{2}}I_{N}. \end{array}$$

In a similar fashion to sampling $o_{1}$, sampling $o_{2}$ reduces to sampling from a multivariate Gaussian distribution with the following mean and covariance:

(A22) $$\begin{array}{c} \mu_{o_{2}}=\frac{\alpha^{2}}{\rho^{2}+\alpha^{2}}\left(Zt-c\right)\;\mathrm{and}\;\;\;\;\Sigma_{o_{2}}=\frac{\rho^{2}\alpha^{2}}{\rho^{2}+\alpha^{2}}I_{N}. \end{array}$$

Algorithm A1 is a detailed version of Algorithm 1 following the sampling steps explained above. As $t_{z_{g}=0}$ generated in step (a) is not used during the following steps, it can thus be omitted.

**Algorithm A1** Split and augmented—partially collapsed Gibbs sampling algorithm for activity estimation in PGET—version II.
1: **Fixed input parameters**: Number of burn-in iterations $N_{\mathrm{bi}}$; total number of iterations $N_{\mathrm{MC}}$ 2: **Initialization** (k=0) • Set $z^{\left(0\right)},t^{\left(0\right)},b^{\left(0\right)},c^{\left(0\right)}$, $\omega^{\left(0\right)},{s^{2}}^{\left(0\right)}$, $o_{1}^{\left(0\right)},o_{2}^{\left(0\right)}$ 3: **Repeat ($1\leq k\leq N_{\mathrm{MC}}$)** (1a) Sample ${s^{2}}^{\left(k\right)}|\left(y,\omega^{\left(k-1\right)},\Delta_{\setminus t_{z_{g}=0}}^{\left(k-1\right)},\Upsilon^{\left(k-1\right)},\Lambda^{\left(k-1\right)}\right)$ from Equation (A4). (2a) Sample $z^{\left(k\right)}|\left(y,t^{\left(k\right)},{s^{2}}^{\left(k\right)},\omega^{\left(k-1\right)},\Upsilon^{\left(k-1\right)},\Lambda^{\left(k-1\right)}\right)$ from Equation (A8). (2b) Sample $t^{\left(k\right)}|\left(y,z^{\left(k\right)},{s^{2}}^{\left(k\right)},\omega^{\left(k-1\right)},\Upsilon^{\left(k-1\right)},\Lambda^{\left(k-1\right)}\right)$ from Equation (A9). (3) Sample $\omega^{\left(k\right)}|\left(y,{s^{2}}^{\left(k\right)},\Delta^{\left(k\right)},\Upsilon^{\left(k-1\right)},\Lambda^{\left(k-1\right)}\right)$ from Equation (A10). (4) Sample $b^{\left(k\right)}|\left(y,\Phi^{\left(k\right)},\Delta^{\left(k\right)},c^{\left(k-1\right)},\Lambda^{\left(k-1\right)}\right)$ from Equation (A14). (5) Sample $c^{\left(k\right)}|\left(y,\Phi^{\left(k\right)},\Delta^{\left(k\right)},b^{\left(k\right)},\Lambda^{\left(k-1\right)}\right)$ from Equation (A9). (6) Sample $o_{1}^{\left(k\right)}|\left(y,\Phi^{\left(k\right)},\Delta^{\left(k\right)},\Upsilon^{\left(k\right)},o_{2}^{\left(k-1\right)}\right)$ from Equation (A20). (7) Sample $o_{2}^{\left(k\right)}|\left(y,\Phi^{\left(k\right)},\Delta^{\left(k\right)},\Upsilon^{\left(k\right)},o_{1}^{\left(k\right)}\right)$ from Equation (A22). 4: **Set**k=k+1.
